# Political and public acceptability of a sugar-sweetened beverages tax: a mixed-method systematic review and meta-analysis

**DOI:** 10.1186/s12966-019-0843-0

**Published:** 2019-09-04

**Authors:** Michelle Eykelenboom, Maartje M. van Stralen, Margreet R. Olthof, Linda J. Schoonmade, Ingrid H. M. Steenhuis, Carry M. Renders, on behalf of the PEN Consortium, Joerg Meerpohl

**Affiliations:** 1 Department of Health Sciences, Faculty of Science, Vrije Universiteit Amsterdam, and Amsterdam Public Health Research Institute, Amsterdam, the Netherlands; 2 Medical Library, Vrije Universiteit Amsterdam, Amsterdam, the Netherlands

**Keywords:** Acceptability, Nutrition policy, Obesity prevention, Public opinion, Public support, Sugar-sweetened beverages, Taxes

## Abstract

**Background:**

Taxation of sugar-sweetened beverages (SSBs), as a component of a comprehensive strategy, has emerged as an apparent effective intervention to counteract the rising prevalence of overweight and obesity. Insight into the political and public acceptability may help adoption and implementation in countries with governments that are considering an SSBs tax. Hence, we aimed to conduct a systematic review and meta-analysis to synthesize the existing qualitative and quantitative literature on political and public acceptability of an SSBs tax.

**Methods:**

Four electronic databases (PubMed, Embase, Scopus, Web of Science) were searched until November 2018. The methodological quality of the included studies was assessed using the Mixed Methods Appraisal Tool. Qualitative studies were analyzed using a thematic synthesis. Quantitative studies were analyzed using a random-effects meta-analysis for the pooling of proportions.

**Results:**

Thirty-seven articles reporting on forty studies were eligible for inclusion. Five themes derived from the thematic synthesis: (i) beliefs about effectiveness and cost-effectiveness, (ii) appropriateness, (iii) economic and socioeconomic benefit, (iv) policy adoption and implementation, and (v) public mistrust of the industry, government and public health experts. Results of the meta-analysis indicated that of the public 42% (95% CI = 0.38–0.47) supports an SSBs tax, 39% (0.29–0.50) supports an SSBs tax as a strategy to reduce obesity, and 66% (0.60–0.72) supports an SSBs tax if revenue is used for health initiatives.

**Conclusions:**

Beliefs about effectiveness and cost-effectiveness, appropriateness, economic and socioeconomic benefit, policy adoption and implementation, and public mistrust of the industry, government and public health experts have important implications for the political and public acceptability of an SSBs tax. We provide recommendations to increase acceptability and enhance successful adoption and implementation of an SSBs tax: (i) address inconsistencies between identified beliefs and scientific literature, (ii) use raised revenue for health initiatives, (iii) communicate transparently about the true purpose of the tax, and (iv) generate political priority for solutions to the challenges to implementation.

**Electronic supplementary material:**

The online version of this article (10.1186/s12966-019-0843-0) contains supplementary material, which is available to authorized users.

## Background

Consumption of sugar-sweetened beverages (SSBs) has been identified as an important modifiable risk factor for overweight and obesity [1]. It has been estimated that SSBs account for at least one-fifth of the weight gained between 1977 and 2007 in the United States (US) population [2]. Therefore, SSBs are a target for many obesity prevention interventions [1]. Results from large prospective cohort studies and randomized controlled trials (RCTs) indicate that SSB consumption promotes weight gain in both children and adults [3]. RCTs in children showed a 0.12 to 0.17-unit reduction in body mass index gain when SSBs were reduced, whereas RCTs in adults showed a 0.85 to 1.20 kg increase in body weight when SSBs were added [3]. Potential underlying causes include their high levels of added sugar, low satiety and incomplete compensation for energy consumed, which can result in a positive energy balance and subsequent weight gain [1]. Furthermore, SSB consumption is associated with an increased risk of type 2 diabetes, cardiovascular disease and dental caries [1].

Globally, the consumption of SSBs is increasing steadily [4]. In recent years, SSB consumption is rising fastest in regions of the world beyond high income countries, many of which are low and middle income countries (e.g. China, Thailand, Brazil and Chile) [3, 4]. SSBs are the largest contributor to energy intake [3, 4]. For example, SSBs contribute to 8.0 and 6.9% of daily energy intake among children and adults in the US, respectively [5]. In addition, results from the National Diet and Nutrition Survey 2014/2015–2015/2016 demonstrate that SSBs contribute to a significant proportion of sugar consumed in the United Kingdom (UK), particularly by children and young adults (e.g. up to 33% for children aged 11 to 18 years) [6]. Decreasing SSB consumption could substantially reduce obesity and obesity-related diseases [7].

Taxation of SSBs, as a component of a comprehensive strategy, has emerged as an apparent effective intervention to counteract the rising prevalence of overweight and obesity [8, 9]. Systematic reviews have indicated that an SSBs tax reduces SSB consumption and improves weight outcomes [8, 10–12]. For example, modelling studies have suggested that the prevalence of overweight and obesity decreases about 3% given an SSBs tax of 20% [8]. Therefore, the World Health Organization (WHO) called on governments to implement an SSBs tax [9]. Reviews of modelling studies have suggested that a tax of 10 to 20% would be necessary to have significant impact on purchases, consumption and ultimately population health [11, 13].

Several countries (e.g. Ireland, Portugal, South Africa and Thailand) and US cities (e.g. Berkeley, California and Philadelphia, Pennsylvania) have introduced taxes on SSBs [14, 15]. However, despite the WHO recommendation and growing evidence suggesting that the taxation of SSBs has the potential to improve health, no such policy has yet been introduced in other countries [16]. In the decision-making process for an SSBs tax, political and public acceptability of the tax are important dimensions as low political and public support may complicate its adoption and implementation [17, 18]. Thus, it is necessary to understand which factors play a role in the political and public acceptability of an SSBs tax. Such an understanding lead to greater insight into arguments used to justify both support and opposition of the tax which, in turn, could inform strategies to enhance acceptability and ultimately improve population health. However, to our knowledge there has been no systematic investigation of political and public acceptability of an SSBs tax.

The present study aimed to perform a systematic review and meta-analysis to synthesize the existing qualitative and quantitative literature on the political and public acceptability of an SSBs tax. Specifically, it intended to: (i) explore political (e.g. policy-makers, politicians and officials of ministries) and public acceptability of an SSBs tax in-depth through qualitative synthesis, and (ii) estimate acceptability of an SSBs tax quantitatively.

## Methods

This review is reported according to the Preferred Reporting Items for Systematic reviews and Meta-Analyses guidelines [19]. The review protocol was registered with PROSPERO, the International Prospective Register of Systematic Reviews (CRD42018090721).

### Search strategy

A comprehensive search was performed from inception to November 14th 2018 in collaboration with a medical librarian (LS). The following electronic databases were used: PubMed, Embase, Scopus and Web of Science. The main key words used in the search strategy were related to three key concepts: ‘SSBs’, ‘tax’ and ‘acceptability’, and were customized to each database. The search was performed without date or language restriction. The search strategy is detailed in Additional file 1: Tables S1a to S1d. Additional searches were conducted by scanning reference lists of identified reviews, meta-analyses and included studies.

### Eligibility screening

Studies were eligible for inclusion if they met the following criteria: (i) the study measured political and/or public acceptability of an SSBs tax, which was defined as the degree to which an SSBs tax is perceived as appropriate, fair and reasonable [20], (ii) the population of the study composed of any individuals involved in the decision-making process (e.g. policy-makers, politicians and officials of ministries) or any individuals potentially affected by an SSBs tax (i.e. the public), and (iii) it was a qualitative, quantitative or mixed-method study of any type. Studies were excluded for the following reasons: (i) the study measured political and/or public acceptability of a sugar tax (i.e. only studies investigating acceptability of a tax specific for SSBs were included), (ii) the study was not published in English, (iii) the study did not contain original data (e.g. editorials, letters to the editor, commentaries, opinion pieces, policy briefs, systematic reviews and meta-analyses), (iv) the study assessed price increases at a non- national or non-state level (e.g. interventions in a school or university setting).

The decision to include studies was hierarchical and initially made on the basis of the study title, followed by the study abstract and finally the full-text article. The selection of studies was independently performed by two reviewers (ME and MS) and any disagreement was discussed until consensus was reached. If no consensus was reached, a third reviewer (CR) was consulted.

### Quality appraisal

The methodological quality of the included studies was assessed using the Mixed Methods Appraisal Tool (MMAT), a valid and reliable tool designed for use in systematic mixed studies reviews (see Additional file 1: Table S2a) [21–23]. The MMAT includes criteria for qualitative studies and for three quantitative study designs: randomized controlled trials, non-randomized studies and descriptive studies. In this review, the appraisal criteria for descriptive studies were used for all included quantitative studies, as only descriptive data were extracted. The quality appraisal was independently performed by two reviewers (ME and MS) and any disagreement was resolved by consensus. If no consensus was reached, a third reviewer (CR) was consulted. The results of the quality assessment were narratively incorporated into the synthesis process.

### Data extraction and synthesis

Data were extracted by two reviewers (ME and MS) and disagreement was discussed until consensus was reached. The following data were extracted from the included studies by using a standardized data extraction form: first author, year of publication, country, taxation at the time of study, study design, data collection method, sample, recruitment/setting and sample size. A sequential exploratory synthesis design was selected to integrate qualitative and quantitative data on political and public acceptability of an SSBs tax. In a sequential exploratory synthesis design, the qualitative synthesis is followed by, and informs, the quantitative synthesis (phase one), and the quantitative synthesis generalizes or tests findings of the qualitative synthesis (phase two) [23]. In this review, the purpose of the sequential exploratory synthesis was to explore political and public acceptability of an SSBs tax (phase one) and to estimate the magnitude of acceptability of an SSBs tax and of arguments related to this acceptability quantitatively (phase two). Interpretation of both phases revealed knowledge gaps and suggested recommendations for policy adoption and implementation.

#### Qualitative studies

For the included qualitative studies, all text in the “Results” or “Findings” section of the article was copied. Data were analyzed in three stages using thematic synthesis [24]. First, all text was coded line-by-line using inductive coding. Second, codes were categorized by similarity to develop descriptive themes that remained close to the included studies. Third, analytical themes were generated in a stage of interpretation [24]. As some qualitative studies included data outside the scope of this review, text lines in which findings were not related to political and/or public acceptability of an SSBs tax were not included in the coding. All stages of the thematic synthesis were carried out independently by two reviewers (ME and MS). Themes identified by the two reviewers were discussed in several meetings until consensus was reached. If no consensus was reached, a third reviewer (CR) was consulted.

#### Quantitative studies

For the included quantitative studies, all measures of public acceptability of an SSBs tax were extracted using predefined criteria (see Additional file 1: Table S3). Measures included proportions of participants reporting support for an SSBs tax and proportions of participants reporting agreement with arguments used to justify both support for and opposition to an SSBs tax. For the purpose of this review, only baseline data of control conditions were extracted from pre-test post-test studies. If relevant data were not reported, authors were contacted to request data (*n* = 10). Previous research indicates that question wording may affect responses [25]. Therefore, extracted measures of support for an SSBs tax were independently grouped into categories by ME and MS based on question wording. Categories identified by the two reviewers were discussed in several meetings until consensus was reached. In addition, extracted measures of arguments used to justify support for or opposition to an SSBs tax were grouped into categories using the themes that emerged from the qualitative synthesis.

Pooling of proportions within each category was performed in Stata using ‘Metaprop’ [26]. Only categories for which data were available from two or more studies were included in meta-analysis. Where categories included more than one measure from the same study, the mean proportion across measures was calculated prior to pooling. The Freeman-Tukey double arcsine transformation was used to stabilize variances. Heterogeneity across studies was assessed using the I2 statistic [27].

## Results

### Literature search

**Fig. 1:**
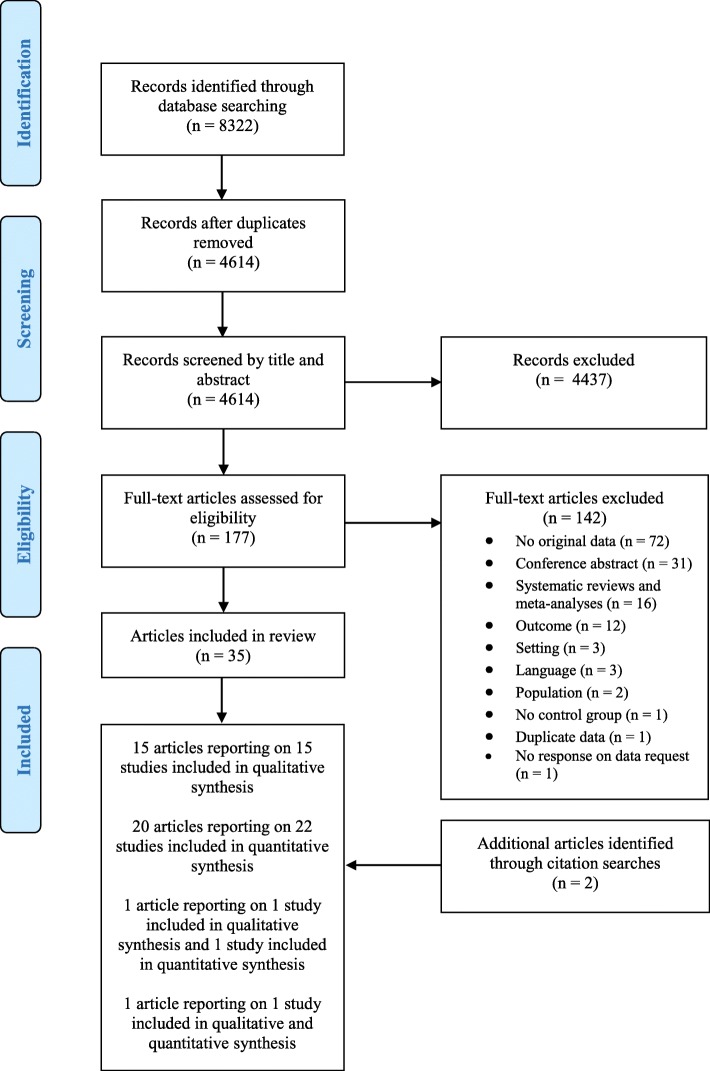
PRISMA flowchart

The search yielded a total of 8322 records (Fig. 1). After removal of duplicates, 4614 records were screened by title and abstract. Of the 177 full-text articles that were assessed for eligibility, 142 were excluded for the reasons as described in Fig. 1. An additional two articles were identified in citation searches, resulting in a total of thirty-seven articles being included in this review reporting on sixteen qualitative studies, twenty-three quantitative studies and one mixed-method study (Fig. 1).

Of the included studies, the majority of studies (*n* = 32) investigated public acceptability of an SSBs tax (e.g. in samples that consisted of adults or students), three studies investigated political acceptability of an SSBs tax (e.g. in samples that consisted of policy-makers, politicians, councilpersons and key informants from the Ministry of Health), and the remaining five studies investigated both political and public acceptability of an SSBs tax. Studies were conducted in the US (*n* = 19), Australia (*n* = 7), the UK (*n* = 5), Mexico (*n* = 3), China (*n* = 1), France (*n* = 1), Israel (*n* = 1), New Zealand (*n* = 1), four Pacific countries (*n* = 1) and fourteen European counties (*n* = 1). Detailed characteristics of the included studies are presented in Table 1.

**Table 1:** Characteristics of included studies on political and public acceptability of a sugar-sweetened beverages (SSBs) tax (*n* = 40)

Author (year)	Country	Taxation at time of study	Study design	Data collection method	Sample^a^		Recruitment/setting	Sample size
Isett (2015) [28]	US	No	Qualitative	Semi-structured interviews	Political and public	Individuals involved in the SSBs policy initiative (past and present public officials, key advocates from the nonprofit sector^b^)	New York City	27 (6^b^)
Moise (2011) [29]	Mexico	No	Qualitative	Semi-structured interviews	Political and public	Key informants (from Ministry of Health and civil society^b^)	Purposive sample	16 (3^b^)
Nixon (2015) [30]	US	No	Qualitative	News reports	Political and public	Published between Nov 2011 and Jan 2013 (Richmond and El Monte) and Nov 2012 and Jan 2014 (Telluride) on soda tax initiatives	Nexis news database and online archives of news sources, Richmond, El Monte and Telluride	378
Signal (2018) [31]	New Zealand	No	Qualitative	Semi-structured interviews	Political and public	Key stakeholders (politicians, bureaucrats and consumer representatives^b^)	Purposive sample	22 (8^b^)
Tamir (2018) [32]	Israel	No	Qualitative	Semi-structured interviews	Political and public	Stakeholders (legislators, policy makers, regulators and public representatives^b^)	Purposive sample	39 (17^b^)
Lloyd-Williams (2014) [33]	Fourteen European countries^d^	Yes in Denmark, Finland, France and Hungary	Qualitative	Semi-structured interviews	Political	National experts from 14 European countries (senior policy-makers^b^)	Purposive sample	71 (12^b^)
Purtle (2018) [34]	US	No	Qualitative	Semi-structured interviews	Political	Key informants closely involved with the SSBs tax policymaking process (city councilpersons, city agency officials^b^)	Philadelphia	9 (6^b^)
Thow (2011) [35]	Four Pacific countries^e^	Yes	Qualitative	Semi-structured interviews	Political	Stakeholders (politicians and policy makers from both health and finance^b^)	Snowball sample, Fiji, Samoa, Nauru and French Polynesia	Not reported
Chan (2009) [36]	China	No	Qualitative	Focus groups	Public	Eighth and ninth grade adolescents (13–15 years)	Purposive sample, Hong Kong	22
Francis (2017) [37]	Australia	No	Qualitative	Group interviews	Public	Young people (12–25 years)	Convenience sample of young people attending one youth group and two high schools, Perth	41
Giabbanelli (2016) [38]	US	Pre- and post-implementation period	Qualitative	Reader comments	Public	Comments on SSBs taxes in Berkeley and San Francisco in news reports published between 1 Jan 2014 and 31 Jan 2015	LexisNexis database, large U.S. daily newspapers and newspapers with significant readership in Berkeley or San Francisco, California	3864
Krukowski (2016) [39]	US	No	Qualitative	Focus groups	Public	Students in grades six through eight	Random sample from a middle school, Michigan	22
Ortega-Avila (2018) [40]	Mexico	Yes	Qualitative	Semi-structured interviews	Public	Adolescents (15–19 years)	Purposive sample recruited through participation in an earlier cross-sectional survey, north-west Mexico	29
Swift (2018) [41]	UK	No	Qualitative	Forum posts	Public	Forum posts that referred to the proposed Soft Drinks Industry Levy posted between 17 July 2015 and 31 Aug 2016	Top three UK online parenting forums	412
Thomas-Meyer (2017) [42]	UK	No	Qualitative	Reader comments	Public	To online articles published on 10 UK news websites with the most total unique visitors in Jan 2013	Popular online news websites	1645
Visram (2017) [43]	UK	No	Qualitative	Focus groups	Public	Pupils from year 6 (10–11 years) and year 9 (13–14 years)	Four schools in County Durham, northern England	37
Moretto (2014) [44]	Australia	No	Mixed-method	Citizens’ Jury	Public	Adults (≥ 18 years)	Purposive sample of citizens randomly selected from the electoral roll, Queensland	13
Álvarez-Sánchez (2018) [45]	Mexico	Yes	Cross-sectional survey	Self-reported questionnaire administered face-to-face	Public	Adults (20–59 years)	Nationally representative sample of residents, the 2016 Mexican National Health and Nutrition Survey	6650
Barry (2013) [25]	US	No	Cross-sectional survey	Online self-report questionnaire	Public	Adults (18–64 years)	Probability-based sample from the nationally representative GfK	1026
Brock (2017) [46]	US	No	Cross-sectional survey	Questionnaire administrated via phone	Public	Adults	Stratified probability sample randomly selected at household level from the 2014 Texas Lyceum Poll, Texas	575
Comans (2017) [47]	Australia	No	Cross-sectional survey	Online self-report questionnaire	Public	Parents/caregivers of young children (3–7 years)	Subsample from the Environments for Health Living cohort study, South-East Queensland	563
Curry (2018) [48]	US	No	Cross-sectional survey	Questionnaire administrated via phone	Public	Adults (≥ 18 years)	Representative random sample of residents, Kansas	2203
Donaldson (2015) [49]	US	No	Cross-sectional survey	Questionnaire administrated via phone	Public	Adults (≥ 18 years)	Randomly selected representative sample of registered voters, Mid-Atlantic state	1000
Farrell (2018) [50]	Australia	No	Cross-sectional survey	Interview questionnaire administered face-to-face	Public	Children and adults (≥ 15 years)	Representative sample of residents, the 2014 South Australian Health Omnibus Survey, South-Australia	2732
Gollust (2014) [51]	US	No	Cross-sectional survey	Online self-report questionnaire	Public	Adults (18–64 years)	Nationally representative sample recruited from a GfK panel	1319
Gollust (2017) [52]	US	No	Cross-sectional survey	Online self-report questionnaire	Public	Undergraduate students	Large university, Minnesota	494
Julia (2015) [18]	France	Yes	Cross-sectional survey	Online self-report questionnaire	Public	Adults (≥ 18 years)	Nationally representative stratified random subsample from the Nutrinet-Santé cohort study	1996
Morley (2012) [53]	Australia	No	Cross-sectional survey	Questionnaire administrated via phone	Public	Adults (18–64 years) who were the main grocery buyer for their household	Random sample	1521
Petrescu (2016) [54]	US	No	Cross-sectional survey	Online self-report questionnaire	Public	Adults (≥ 18 years)	Sample recruited using Amazon Mechanical Turk	1082
Petrescu (2016) [54]	UK	No	Cross-sectional survey	Online self-report questionnaire	Public	Adults (≥ 18 years)	Sample recruited by Survey Sampling International	1093
Rivard (2012) [55]	US	No	Cross-sectional survey	Questionnaire administrated via phone	Public	Adults (≥ 18 years)	Representative sample recruited by the Survey Research and Data Acquisition Resource	592
Roh (2016) [56]	US	No	Cross-sectional survey	Online self-report questionnaire	Public	Adults (≥ 18 years)	Web participants recruited from Amazon.com’s Mechanical Turk	206
Roh (2016) [56]	US	No	Cross-sectional survey	Questionnaire administrated via phone	Public	Adults (≥ 18 years)	Probability sample recruited from a Marketing Systems Group’s national panel	1000
Sainsbury (2018) [57]	Australia	No	Cross-sectional survey	Online self-report questionnaire	Public	Adults (≥ 18 years)	Nationally representative sample recruited by Online Research Unit	2011
Simon (2014) [58]	US	No	Cross-sectional survey	Questionnaire administrated via phone	Public	Adults (≥ 18 years)	Random subsample from the 2011 Los Angeles Country Health Survey, Los Angeles	998
Swift (2018) [41]	UK	No	Cross-sectional survey	Online self-report questionnaire	Public	Adults (≥ 18 years)	Advertised on four popular parenting forums	184
Tabak (2013) [59]	US	No	Cross-sectional survey	Questionnaire administrated via phone	Public	Adults (≥ 18 years)	Random sample, Mississippi	2800
Wolfson (2015) [60]	US	No	Cross-sectional survey	Online self-report questionnaire	Public	Adults (18–64 years)	Probability-based sample recruited using the GfK survey research panel, Policy Support Survey	408
Niederdeppe (2014) [61]	US	No	Pre-test post-test	Self-reported questionnaire (method of administration not reported)	Public	Adults (18–64 years)	Probability sample from the GfK panel	3118 (941^c^)
Scully (2017) [62]	Australia	No	Pre-test post-test	Online self-report questionnaire	Public	Adults (≥ 18 years)	Random sample of panel members from two non-probability based online panels	6000 (300^c^)

^a^ Political, any individuals involved in the decision-making process (e.g. policy-makers, politicians and informants from ministries); public, any individuals potentially affected by an SSBs tax (i.e. the public); ^b^ Participants representative of the population of interest; ^c^ Participants assigned to the control condition; ^d^ Fourteen European countries: Belgium, Czech Republic, England, Estonia, Finland, Germany, Greece, Iceland, Italy, Ireland, Malta, Poland, Portugal and Slovenia; ^e^ Four Pacific countries: Fiji, Samoa, Nauru and French Polynesia

### Quality appraisal

All included studies reported clear research questions or objectives, and in all studies the collected data allow to address the research questions or objectives. Details of the quality appraisal are shown in Additional file 1: Tables S2b and S2c.

All qualitative studies used sources of data that were relevant to address the research questions or objectives (i.e. participants, reader comments and news reports), and used a relevant method of data collection (e.g. semi-structured interviews, focus groups and online data sources) and analysis (e.g. thematic analysis and content analysis). In addition, all qualitative studies described the context in which data were collected. Thirteen of the seventeen studies included in the qualitative synthesis described the researchers’ role at all stages.

Eighteen of the twenty-four studies included in the quantitative synthesis were judged to have an adequate random sampling strategy, three studies an inadequate sampling strategy, and the remaining studies an unclear sampling strategy. The majority of studies (*n* = 17) were judged to have representative samples. All studies used clearly defined measures for acceptability of an SSBs tax. Two studies had an acceptable response rate of ≥60%.

### Synthesis of qualitative studies

Five themes derived from the thematic synthesis of the qualitative studies: (i) beliefs about effectiveness and cost-effectiveness, (ii) beliefs about appropriateness, (iii) beliefs about economic and socioeconomic benefit, (iv) beliefs about policy adoption and implementation, and (v) public mistrust of the industry, government and public health experts. Themes and subthemes with illustrative quotes are presented in Table 2. Fewer subthemes were observed in studies with taxation at the time of study [35, 40] than in studies without taxation (e.g. the subthemes ‘cost-effectiveness’, ‘negative economic impact’, ‘socioeconomic equality’, ‘availability of healthy alternatives’, and the theme ‘mistrust’ did not derive from studies with taxation). However, the beliefs within subthemes that were observed in studies with taxation at the time of study as well as in studies without taxation were the same, regardless of current taxation.

**Table 2:** Synthesis of qualitative studies on political and public acceptability of a sugar-sweetened beverages (SSBs) tax

Theme	Subtheme	Studies on political acceptability	Studies on public acceptability	Illustrative quotes
Beliefs about effectiveness and cost-effectiveness	Impact on SSB purchases and consumption	[28–30, 32, 35]^c^	[32, 36–43]^c^	‘Informants from academia and MOH [Ministry of Health] indicated that taxation may limit SSB consumption’ (Mexico) [29] ‘Many students stated that a positive of SSB taxes was decreased consumption (“I think it would stop so many people from buying sugary drinks.”)’ (US) [39] ‘One female participant said that people had already made up their minds about soft drinks. Those who really love soft drinks would save money from other sources to buy them anyway’ (China) [36] ‘Another student concluded, “I think that the only people that will actually do that [continue to buy SSBs] are people that are really rich and can afford anything or people that just don’t know how to handle an addiction of theirs.”’ (US) [39] ‘A lack of awareness of the price of beverages was reported as a reason for dismissing taxation, as one participant explained: It won’t affect me [tax] because, like me, other people don’t remember the juice prices of last year’ (Mexico) [40]
	Impact on health-related outcomes	[28, 30, 31, 33–35]^c^	[28–31, 38, 41, 42]	‘The Minister for Health proposed the tax due to concerns over diabetes and other chronic diseases’ (Pacific countries) [35] ‘Research evidence about the health effects of SSBs and potential health benefits of SSB taxes was influential toward the end of the policymaking process’ (US) [34] ‘A number of stakeholders were awaiting outcomes from the Mexican SSBs tax, which were not published at the time.’ (New Zealand) [31] ‘Tax is a cure for obesity’ (US) [38] ‘Many commenters thought it would not be not effective in reducing obesity’ (UK) [42]
	Impact on SSB prices	[31]	[38–40, 42, 44]^c^	‘There were concerns that the full tax may not be passed on to consumers by companies as they may spread the load across all types of sweetened and non-sweetened drinks’ (Australia) [44] ‘According to several participants, the tax would only affect them if it were higher: ‘If the price was much higher than it was before, I think yes, I would consider it, but the 10% increase is not that much’ (Mexico) [40]
	Availability of healthy alternatives	[29, 31, 32]	[29, 32, 41, 42, 44]	‘All informants emphasized the structural barriers to implementation, including an inadequate investment in drinking water infrastructure and lack of healthy alternatives to SSB’ (Mexico) [29] ‘I would much prefer to feed my children a sugary drink then take them out to play in the park and burn it off then so-called diet drinks filled with neurotoxins like Aspartame that does Lord-knows-what to their developing brains’ (UK) [42] ‘I’d rather sugar than artificial sweeteners …’ (UK) [41]
	Encourage industry to reformulate content		[42]	‘It would encourage the drink producers to reduce the sugar’ (UK) [42]
	Cost-effectiveness	[32–34]	[30, 38, 42]	‘Participants across all 14 European countries also perceived regulatory measures to be more cost-effective than voluntary measures’ (European countries) [33] ‘If a tax helps that then so be it, it’s worth the extra cost (and will save us loads in the future)’ (UK) [42]
Beliefs about appropriateness	Taxation as an intervention strategy	[28, 29, 31–33, 35]^c^	[29, 30, 32, 38, 42]	‘To counter marketing practices and SSB consumption, almost all insisted that ‘government intervention is crucial to protect children’ (Mexico) [29] ‘Analogies were made to the successes of tobacco taxes and the transfats restriction in improving health’ (US) [28] ‘But I’m an adult and I’m sick to death of being treated like a 5 year old by this nanny-state we’re now living in’ (UK) [42] ‘*There should be a limit to government intervention, even if the cause is just and for good values. I think there should be limits to what the government is allowed to intervene in’ (Israel)* [32] ‘Bring down the price of healthy foods and then see if there is a decline in obesity’ (UK) [42]
	SSBs as an intervention target	[31–33]	[28–31, 39–44]^c^	‘Four noted that soft drinks are cheaper than healthy alternatives, suggesting that a tax would assist in adjusting this anomaly, including one consumer, one politician, and one food industry leader’ (New Zealand) [31] ‘The jurors agreed that sugar-sweetened drinks were (…) a major contributor to childhood obesity’ (Australia) [44] ‘I’ve drank soda all my life and I’ve never been overweight’ (UK) [42]
	Beliefs about overweight and obesity	[32]	[30, 32, 38, 41, 42]	‘One long-term resident observed, ‘[I’ve] looked at children in the schoolyard and on the streets of Telluride and, for the life of me, I don’t see obesity as a local problem’ (US) [30] ‘If youre fat, its your own fault and its YOUR responsibility to do something about it. Not the NHS Not the Govt Not the tax payer ... Take some responsibility and put down the fork!’ (UK) [42] ‘All of the interviewees, regardless of sector, regarded obesity as a combined public and personal problem’ (Israel) [32]
Beliefs about economic and socioeconomic benefit	Raise revenue for societal health programs	[30, 32, 34, 35]^c^	[30, 32, 38, 42]	‘In 2002 the French Polynesian government introduced a range of taxes, including taxes on soft drinks, in order to fund the establishment of the Etablissement pour la prevention (EPAP), a prevention fund’ (Pacific countries) [35] ‘One local mother described in glowing terms her daughter’s experience, concluding, ‘I am prepared to pay an extra 12 cents for my occasional soda … if it means the continuation of these programs for the children of Telluride’ (US) [30]
	Raise revenue for health care	[28, 35]^c^	[42]	‘The government at the time proposed the tax because they wanted to enact preventive health interventions as well as fund hospitals’ (Pacific countries) [35]
				‘A number of suggestions were made for how tax revenues could be spent to benefit society as a whole–particularly via additional financial support for the NHS’ (UK) [42]
	Raise revenue for the general budget	[28, 30, 32, 35]^c^		‘Mayor Andre Quintero said, ‘[T] here are significant financial hurdles that we need to start dealing with now, so having this type of tax as an option brings in revenue’ (US) [30]
	Negative economic impact	[31, 32]	[32, 38, 39]	‘One bureaucrat noted the value of evidence of the impact on the economy and productivity’ (New Zealand) [31] ‘The corporations absorb the hit and reduce jobs to offset the increased cost of doing business’ (US) [38]
	Impact on socioeconomic equality	[31–34]	[28, 30–32, 38, 41, 42]	‘Pro-SSB tax advocates, including the LHD, countered this argument with data about the disproportionally high prevalence of obesity and diabetes among low-income Philadelphians and claims that these disparities were regressive’ (US) [34] ‘The main arguments were the regressive nature of the tax - not only would low-income families be spending more of their income on the tax, but these communities also consume greater quantities of soda than higher-income populations’ (US) [28]
Beliefs about policy adoption and implementation	Feasibility	[29, 31–33, 35]^c^	[29, 31, 32, 41, 42, 44]	‘There was general agreement that a soft drink tax would be the easiest intervention to implement of the ones examined. It was agreed that it would be possible to impose an excise tax, such as that on alcohol, as occurs in New Zealand’ (New Zealand) [31] ‘The jurors agreed that sugar-sweetened drinks were easily defined’ (Australia) [44] ‘When asked about the legal framework, informants described a convoluted policymaking system, a prolonged policy adoption process, competing agendas, and opposition (mostly from industry)’ (Mexico) [29] ‘Five people commented on the administrative load of such a tax. A bureaucrat and food industry leader were concerned the load would be high’ (New Zealand) [31] ‘Policymakers are fighting ‘an invisible enemy’ in home-made, unlabeled products, including home-made sugary beverages, that will be difficult to regulate’ (Mexico) [29]
	Support of stakeholders	[29–32, 35]^c^	[29, 31, 32, 39, 42]	‘There was agreement from all stakeholder groups on the need to increase public support for such a tax’ (New Zealand) [31] ‘Regarding people such as doctors, whom students believed would be against adolescents’ drinking SSBs, they stated, “They would think that it [a SSB tax] is a good idea.”’ (US) [39] ‘Other potential objectors identified by interviewees were members of the tax system: the Ministry of Finance and the Israeli Tax Authority, as well as Members of Parliament and liberal organizations’ (Israel) [32]
				‘However, a consumer representative warned about the power of industry, ‘the hugely influential weight of the industry, you know, they’re in there lobbying the government all the time’ (New Zealand) [31]
Mistrust	Mistrust of industry		[30, 31, 39, 41, 42]	‘Soda companies ‘want to avoid paying their fair share and don’t care about the safety and health of El Monte’s neighborhoods’ (US) [30]
	Mistrust of government	[32]	[30, 38, 41, 42]	‘Some consumers (…) have doubts about the use of proceeds (e.g., “revenue to fund other projects or even their own generous pay raises”)’ (US) [38] ‘… as others have said a tax on sugary drinks is just a government money generating scheme and not addressing the real issues …’ (UK) [41] ‘We know that the Ministry of Finance does not like the use of tax money for a specific cause. The Ministry of Finance wants every penny they collect to be free for use towards the purposes that they choose’ (Israel) [32]
	Mistrust of public health experts		[42]	‘How many of these “experts” struggle with their grocery bill? I‘m sick and tired of hearing “experts” calling for a rise in the cost of living’ (UK) [42]

^a^ Four Pacific countries: Fiji, Samoa, Nauru and French Polynesia; ^b^ Fourteen European countries: Belgium, Czech Republic, England, Estonia, Finland, Germany, Greece, Iceland, Italy, Ireland, Malta, Poland, Portugal and Slovenia; ^c^ Subtheme includes a study with taxation at the time of study

#### Beliefs about effectiveness and cost-effectiveness

##### Impact on SSB purchases and consumption

The belief that an SSBs tax would be effective in reducing purchases and consumption of SSBs was reported in studies on political acceptability [28–30, 35], and in studies on public acceptability [36–43]. This belief generally arose from the belief that ‘price is an important factor in purchase decisions’ [36]. One study on political acceptability reported doubts about the effect on reducing consumption among Israeli legislators, policy makers and regulators [32]. In addition, some participants in studies on public acceptability felt that increased SSB prices as result of a tax would not impact SSB purchases and consumption [32, 36, 38–43]. In particular, an SSBs tax was perceived to be ineffective in those addicted to SSBs [36, 39, 40, 42], in those who lacked awareness of SSB prices [38, 40, 42], in those with obesity, and in rich and stubborn people [39].

##### Impact on health-related outcomes

Overall, studies on political acceptability indicated that an SSBs tax was perceived to be effective in improving health-related outcomes, such as obesity and diabetes [28, 30, 31, 33–35]. This tended to relate to beliefs about scientific evidence on the detrimental health effects of SSBs and the beneficial health effects of an SSBs tax [28, 31, 34]. Some decision-makers in Philadelphia and New Zealand however felt that more scientific evidence could be useful [31, 34]. More reservations about the effectiveness of the tax in improving health-related outcomes were observed in studies on public acceptability. While some studies among the public reported the belief that an SSBs tax could improve population health [30, 31, 38, 41, 42], others indicated that such a policy does not cure anything [38, 41, 42].

##### Impact on SSB prices

Concerns about the impact of an SSBs tax on SSB prices were reported in six studies on political [31] and public acceptability [38–40, 42, 44]. These concerns arose from the belief that an SSBs tax may not be passed through to consumers because of interference by the industry and vendors of SSBs [31, 38, 42, 44]. Minimum prices were suggested by bureaucrats from New Zealand to avoid this potential problem [31]. In addition, some felt that the proposed tax rate was too low to have substantial impact on SSB prices [39, 40, 42, 44]. Studies among Australian citizen jurors and students from Michigan, UK, indicated that a tax rate of 50 to 100% may be large enough to change consumer behavior [39, 44].

##### Encourage industry to reformulate content

One study on public acceptability reported the belief that an SSBs tax would encourage the SSB industry to reformulate SSB content [42]. UK news website commentators indicated that manufacturers would reduce the amount of sugar as a consequence of the tax, which was viewed as a potential facilitator in the effectiveness of an SSBs tax [42].

##### Availability of healthy alternatives

The subtheme availability of healthy alternatives was identified in six studies on political [29, 31, 32] and public acceptability [29, 32, 41, 42, 44], and related to the subtheme ‘feasibility of implementation’. For example, informants from Mexico indicated that taxation was not feasible because of an ‘inadequate investment in drinking water infrastructure’ [29]. Three studies on public acceptability reported concerns about an increase in the consumption of artificial sweeteners as a result of an SSBs tax [41, 42, 44]. To encourage substitution of SSBs with healthy alternatives, Australian jurors suggested to reduce the price of ‘packaged unflavored water’ [44].

##### Cost-effectiveness

An SSBs tax was seen as a cost-effective intervention for improving public health nutrition and obesity prevention across six studies on political [32–34] and public acceptability [30, 38, 42]. For example, senior food policy makers from fourteen European countries perceived regulatory measures to be more cost-effective for improving public health nutrition than voluntary measures [33]. In addition, UK news website commentators believed that an SSBs tax would be ‘worth the extra costs’, because it will save costs in the future [42].

#### Beliefs about appropriateness

##### Taxation as an intervention strategy

Taxation was viewed as an appropriate intervention strategy in the majority of studies on political acceptability [28, 29, 31, 33, 35]. An argument used to justify appropriateness in studies conducted in Mexico and European countries was the perceived need for government intervention to counter SSB consumption [29, 33]. Regulatory instruments, including taxation, were mentioned as appropriate policy tools [29]. In addition, decision-makers referred to the use of taxation on tobacco and alcohol [28, 31, 35]. Existence and successes of these comparable taxes contributed to the belief that taxation is an appropriate intervention strategy to reduce usage of these products. Taxation was also considered necessary in two studies on public acceptability [29, 42]; for example, some UK news website commentators argued that such a policy is needed when individuals are ‘unable to take responsibility for their own behavior’ [42]. However, in other studies on political [32] and public acceptability [30, 32, 38, 42] taxation was viewed as government intrusion. Furthermore, a UK news website commentator indicated that subsidies would be more appropriate and suggested to ‘down the price of healthy foods’ [42].

##### SSBs as an intervention target

SSBs were perceived as an appropriate intervention target in two studies on political acceptability conducted among senior policy-makers from fourteen European countries and politicians and bureaucrats from New Zealand [31, 33]. For example, a politician from New Zealand indicated that ‘soft drinks are cheaper than healthy alternatives’ [31]. However, in one study on political acceptability Israeli regulators perceived SSBs as ‘a source of pleasure’ and therefore felt that ‘taxing them would harm the public’ [32]. Ten studies on public acceptability reported beliefs on the appropriateness of SSBs as an intervention target [28–31, 39–44]. This tended to relate to beliefs about the contribution of SSBs to obesity [28–31, 39, 41, 42, 44], and beliefs about prices of SSBs [31, 43]. Those supportive of an SSBs tax believed that SSBs are a major contributor to obesity [30, 31, 39, 41, 42, 44], while opponents indicated a lack of personal evidence that SSBs can cause obesity and referred to the many other determinants of obesity [28, 29, 41, 42]. Concerning SSB prices, students from County Durham, UK, indicated that some energy drinks were currently cheaper than water [43].

##### Beliefs about overweight and obesity

Five studies indicated that beliefs about overweight and obesity are important in political [32] and public acceptability of an SSBs tax [30, 32, 38, 41, 42]. For example, Telluride residents, US, did not appear to feel that obesity is a local health problem that needs to be addressed and therefore opposed the tax [30]. In addition, some commentators on UK news websites felt that individuals with overweight and obesity are responsible for their own behavior [42]. This belief about the attribution of responsibility for overweight and obesity related to the subtheme ‘taxation as an intervention strategy’, as those commentators perceived an SSBs tax as unfair to ‘healthy’ individuals who consume SSBs responsible [42].

#### Beliefs about economic and socioeconomic benefit

##### Raise revenue for societal health programs

Across six studies on political [30, 32, 34, 35] and public acceptability of an SSBs tax [30, 32, 38, 42], the potential to raise revenue for societal health programs (e.g. for prevention funds, sport fields and recreational activities) was perceived as a positive consequence of implementation.

##### Raise revenue for health care

The potential of an SSBs tax to raise revenue for health care (e.g. for the National Health Service) was identified in three studies on political [28, 35] and public acceptability [42]. For example, governor Patterson of New York, US, argued that the tax could help to ‘defray costs of care for those with obesity’ [28].

##### Raise revenue for the general budget

Four studies on political acceptability reported that an SSBs tax was viewed as a potential to raise revenue for the general budget [28, 30, 32, 35]. For example, the tax was perceived to increase revenue to ‘balance the city budget’ in El Monte, US [30], ‘compensate for losses due to tariff reductions’ in Fiji [35], and compensate for ‘declining phosphate mining income’ in Nauru [35]. In Israel, the ministry of finance was described to be against the use of tax revenue for specific purposes [32].

##### Negative economic impact

Concerns about the negative impact of an SSBs tax on the economy were reported in four studies on political [31, 32] and public acceptability [32, 38, 39], such as concerns about a reduction in jobs and closing of SSB companies as a result of the tax. In addition, a bureaucrat from New Zealand indicated a need for more scientific evidence on the impact of an SSBs tax on economy and productivity [31].

##### Impact on socioeconomic equality

In three studies on political acceptability [31, 33, 34], an SSBs tax was believed to have a positive impact on equality in health. For example, the Local Health Department of Philadelphia, US, argued that an SSBs tax could adjust health disparities. However, in one study on political acceptability conducted in Israel [32] and in the majority of studies on public acceptability [28, 30–32, 38, 41, 42], concerns about the negative impact of an SSBs tax on socioeconomic equality were reported. These concerns primarily arose from the belief that an SSBs tax is regressive [28, 30–32, 38, 41, 42]; low-income individuals have to spend relatively more of their income and consume greater quantities of SSBs [28, 42].

#### Beliefs about policy adoption and implementation

##### Feasibility

Implementation of an SSBs tax was considered feasible in six studies on political [29, 31, 33, 35] and public acceptability [29, 31, 42, 44]. Further, several perceived barriers to the implementation of an SSBs tax were identified. Examples of barriers are a long lawmaking process in Mexico and the UK [29, 41], competing national agendas in Mexico [29], the difficulty of defining products that should be taxed [32, 41] in Israel and the UK, the difficulty of regulating ‘home-made, unlabeled products’ in Mexico [29], the development of a black market in Israel [32], a high administrative load in New Zealand [31], and political costs of taxation in European countries [33].

##### Support of stakeholders

In several studies on political [29–32, 35] and public acceptability [29, 31, 32, 39, 42], beliefs about support of stakeholders were reported. Four stakeholder groups were identified in these studies: the SSB industry (e.g. manufacturers, supermarket chains and catering companies), the public (e.g. consumers), the government (e.g. policy-makers, politicians and ministries) and public health experts (e.g. health professionals and scientific experts) [29–32, 35, 39, 42]. This subtheme overlapped with beliefs about feasibility of implementation of such a policy. A lack of support from these stakeholder groups was identified as a considerable barrier to policy adoption and implementation [29, 31, 32, 35, 42]. Specifically, resistance from the SSB industry was described to complicate policy adoption and implementation. Lobbying of the SSB industry and relationships between the industry and politicians were mentioned in the majority of studies [29–32, 35, 42]. The SSB industry was perceived to have considerable political power. For example, Ministry of Health officials from Mexico pointed out that ‘recent legislative efforts to tax soft drinks have been systematically obstructed’ [29].

#### Mistrust

##### Mistrust of industry

Mistrust of the industry was identified in five studies on public acceptability of an SSBs tax [30, 31, 39, 41, 42]. For example, activities of the SSB industry were criticized in El Monte, US, where the industry was blamed for not caring about safety and health [30].

##### Mistrust of government

Mistrust of the government overlapped with the subthemes regarding the use of raised revenue under the theme ‘Beliefs about economic and socioeconomic benefit’. Public doubts were reported about the use of raised revenue in four studies on public acceptability of an SSBs tax [30, 38, 41, 42]. These doubts tended to relate to doubts about the true purpose of the tax; some UK news website commentators felt that the tax was ‘not truly intended to improve health’ [42]. Furthermore, mistrust of the government was reported in one study on political acceptability of an SSBs tax [32]. Israeli regulators and legislators indicated that the ministry of finance would not use the raised revenue for health purposes [32].

##### Mistrust of public health experts

Mistrust of public health experts was expressed in one study on public acceptability [42]. Some commentators on UK news websites questioned the trustworthiness of the information about SSBs and an SSBs tax provided by public health experts [42].

### Synthesis of quantitative studies

No studies on political acceptability were available for the quantitative synthesis, as the studies we found did not fulfill the inclusion criteria. Therefore, only public acceptability of an SSBs tax is estimated quantitatively.

#### Public acceptability of an SSBs tax

Of the studies included in the quantitative synthesis, nine studies assessed support for an SSBs tax (*n* = 4 in the US, *n* = 3 in Australia, *n* = 1 in France, *n* = 1 in the UK), ten studies assessed support for an SSBs tax as a strategy to reduce obesity (*n* = 8 in the US, *n* = 2 in Australia), and four studies assessed support for an SSBs tax if revenue is appropriately used (i.e. to fund societal health programs) (*n* = 2 in Australia, *n* = 1 in France, *n* = 1 in the US). In addition, thirteen studies assessed agreement with arguments used to justify support for and opposition to an SSBs tax, which were categorized according to the subthemes that emerged from the qualitative synthesis (*n* = 7 in the US, *n* = 2 in Australia, *n* = 1 in France, *n* = 1 in the UK).

**Fig. 2:**
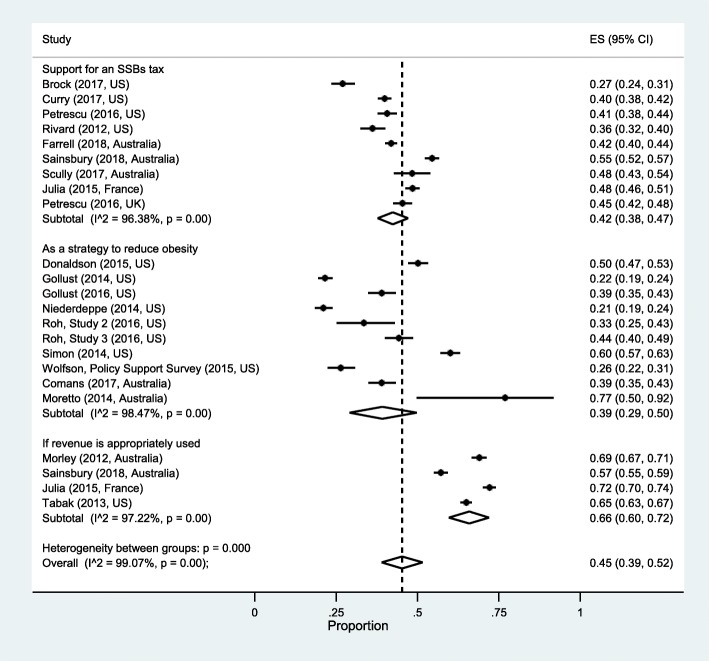
Pooled proportions of public acceptability of a sugar-sweetened beverages (SSBs) tax

Because of significant heterogeneity across studies within categories (*P* < 0.001), data were pooled using a random-effects meta-analysis. Results of the quantitative synthesis of measures of support for an SSBs tax are presented in Fig. 2. Pooled proportions indicated that 42% of the public (95% CI = 0.38–0.47) supports an SSBs tax, 39% of the public (0.29–0.50) supports an SSBs tax as a strategy to reduce obesity, and 66% of the public (0.60–0.72) supports an SSBs tax if revenue is appropriately used (Fig. 2).

Results of the quantitative synthesis of measures of arguments used to justify support for or opposition to an SSBs tax are presented in Table 3. Pooled proportions indicated that of the public 39% (95% CI = 0.26–0.54) believed that an SSBs tax has impact on SSB purchases and consumption, 40% (0.29–0.54) believed that an SSBs tax has impact on health-related outcomes, 68% (0.48–0.85) believed that SSBs are an appropriate intervention target, 92% (0.91–0.93) believed that obesity is a problem, 39% (0.36–0.41) believed that an SSBs tax has the potential to raise revenue for societal health programs, 50% (0.48–0.52) believed that an SSBs tax has a negative impact on socioeconomic equality, 49% (0.32–0.66) mistrusted the industry, and 61% (0.56–0.67) mistrusted the government (Table 3).

**Table 3:** Synthesis of quantitative studies on political and public acceptability of a sugar-sweetened beverages (SSBs) tax

Subtheme	Pooled proportion (95% CI) or proportion	Range	I^2 a^ (%)	No. of studies (references)	Countries
1. Beliefs about effectiveness and cost-effectiveness					
Impact on SSB purchases and consumption	0.39 (0.26–0.54)	0.20–0.64	99.6	7 [18, 25, 45, 49, 54, 55]	France, Mexico, UK, US
Impact on health-related outcomes	0.40 (0.29–0.54)	0.26–0.58	98.5	5 [18, 25, 41, 54]	France, UK, US
Cost-effectiveness	0.33	NA	NA	1 [25]	UK
Lack of healthy alternatives	0.84	NA	NA	1 [45]	Mexico
2. Beliefs about appropriateness					
Taxation as an intervention strategy					
Appropriate	0.37	NA	NA	1 [25]	UK
Not appropriate	0.54	NA	NA	1 [25]	UK
SSBs as an intervention target					
Appropriate	0.68 (0.48–0.85)	0.42–0.92	99.6	5 [25, 45, 49, 52, 55]	Mexico, UK, US
Not appropriate	0.60	NA	NA	1 [25]	UK
Beliefs about overweight and obesity					
Obesity is a problem	0.92 (0.91–0.93)	0.85–0.93	0.00	2 [49, 57]	Australia, US
Society is responsible	0.38	NA	NA	1 [49]	US
3. Beliefs about economic and socioeconomic benefit					
Raise revenue for societal health programs	0.39 (0.36–0.41)	0.37–0.40	0.00	2 [25, 49]	UK, US
Negative economic impact	0.44	NA	NA	1 [25]	UK
Impact on socioeconomic equality					
Negative	0.50 (0.48–0.52)	0.49–0.51	0.00	2 [18, 25]	France, UK
Positive	0.36	NA	NA	1 [25]	UK
4. Beliefs about policy adoption and implementation					
Lack of stakeholder support	0.53	NA	NA	1 [25]	UK
5. Mistrust					
Mistrust of industry	0.49 (0.32–0.66)	0.31–0.80	98.9	4 [51, 52, 61, 62]	UK, US
Mistrust of government	0.61 (0.56–0.67)	0.58–0.67	90.3	3 [25, 54]	UK, US
Mistrust of public health experts	0.35	NA	NA	1 [49]	US

^a^ I^2^: Measure of the degree of inconsistency across studies

## Discussion

The present study is the first systematic review with meta-analysis that synthesized the existing qualitative and quantitative literature on the political and public acceptability of an SSBs tax. We identified thirty-seven relevant articles reporting on forty studies. Five themes derived from the thematic synthesis of the qualitative studies: (i) beliefs about effectiveness and cost-effectiveness, (ii) beliefs about appropriateness, (iii) beliefs about economic and socioeconomic benefit, (iv) beliefs about policy adoption and implementation, and (v) public mistrust of the industry, government and public health experts. Pooled proportions indicated that 39 to 66% of the public supports an SSBs tax, depending on question wording. This review was not able to estimate political acceptability of an SSBs tax, given no quantitative studies on political acceptability did fulfill the inclusion criteria. Four recommendations for policy adoption and implementation are developed based on these findings.

### Address inconsistencies between identified beliefs and scientific literature

It is important to note that several beliefs identified under the themes ‘effectiveness and cost-effectiveness’, ‘appropriateness’ and ‘economic and socioeconomic benefit’ are inconsistent with evidence from scientific literature. For example, the belief that SSB consumption is not a major contributor to obesity is inconsistent with scientific evidence [1–3]. In addition, the belief that an SSBs tax could not impact purchases and consumption of SSBs is not supported by previous research [8, 10–13]. Such inconsistencies may be the result of cognitive dissonance [63]. For example, by believing that SSB consumption is not a major contributor to obesity, consumers of SSBs may reduce the cognitive dissonance that they would have been experienced by consuming SSBs while believing it could contribute to obesity. Furthermore, the inconsistencies between the identified beliefs and scientific literature may be related to mistrust of governments and public health experts as identified in our review or to a lack of knowledge of available scientific literature. The inconsistencies may explain much of the opposition identified among the public and need to be addressed to increase acceptability of an SSBs tax.

### Use raised revenue for health initiatives

The quantitative synthesis of the measures of support revealed that the degree of public acceptability of an SSBs tax in the US, Australia, the UK and France tends to depend on question wording. Public support for an SSBs tax was highest (66%) if revenue is appropriately used. This higher level of support, compared to public support for an SSBs tax and public support for an SSBs tax as a strategy to reduce obesity, could not be explained by differences in year of publication. Based on our findings, public support for an SSBs tax does not seem to be higher in the more recent studies included in our review. However, taxation at the time of study may influence acceptability of an SSBs tax. Public support for an SSBs tax was higher, compared to the pooled proportions, in the quantitative study that investigated acceptability of an SSBs tax after its introduction in France [18]. Although this study was categorized into the “support for an SSBs tax if revenue is appropriately used” category, this may not explain the higher level of support in this category as the other studies in this category showed similar high levels of public support. Therefore, we recommend decision-makers to consider to use revenue for health initiatives to increase public support for an SSBs tax. Furthermore, the finding that public acceptability of an SSBs tax tends to depend on question wording suggests that policy framing strategies could have important implications for the public acceptability of an SSBs tax.

### Communicate transparently about the true purpose of the tax

Our review indicates that more than half of the public (61%) mistrusted the government for not using revenue for health initiatives in studies conducted in the US and the UK. The qualitative synthesis indeed revealed that some decision-makers in Israel, Pacific countries and the US viewed an SSBs tax as an opportunity to raise revenue for the general budget. In agreement with our findings, a previous explorative review by Hagenaars et al. stated that ‘fiscal needs more often seem to lay their policy foundation rather than public health advocacy’ [16]. In several countries the tax rate is smaller than the minimal rate needed to have an impact on purchases, consumption and population health (i.e. 10 to 20%) suggesting that non-health related motives for policy adoption of the tax have played a role [11, 13]. For example, in Fiji there have been an import excise duty of 5% and an excise duty of 5 c/l (US$0.04) on soft drinks [35]. Although we have recommended to use raised revenue for health initiatives rather than for the general budget, in both situations it is important for the government to communicate transparently to the public about the true purpose of the tax in order to prevent public mistrust of the government. Public mistrust has been described to complicate policy implementation and effectiveness [64], which highlights the importance of transparent communication. Noteworthy, our findings indicated that public mistrust of the government (61%) was higher than public mistrust of the industry (49%) in studies conducted in the US and the UK. This difference in public mistrust is not statistically significant and may have been caused by differences in study characteristics, as measures were extracted from different studies. However, it may be interesting to investigate this difference in public mistrust in future studies, because heavily investment of the industry in campaigns that seek to shift away the blame from SSB and create a positive image of the SSBs industry may lower public mistrust of the industry [65].

### Generate political priority for solutions to the challenges to policy adoption and implementation

Our review provides insights into perceived challenges associated with the implementation of an SSBs tax. Several barriers were reported by decision-makers, such as a long lawmaking process in Mexico and the UK, a high administrative load in New Zealand and a lack of support of stakeholders in Israel, Mexico, New Zealand, Pacific countries, the UK and the US. A lack of support of stakeholders was described to complicate policy adoption and implementation of SSBs taxes. In particular, studies referred to resistance from the SSB industry, which seem to have considerable political power. For example, in Mexico the SSB industry systematically obstructed efforts to tax SSBs [29]. Lobbying of the SSBs industry and relationships between the industry and politicians are important barriers that needs to be addressed. The identified challenges may largely explain why difficulties are experienced in countries where an SSBs tax has been introduced, and also why an SSBs tax has not yet been introduced in other countries, despite the general positive beliefs of decision-makers about the effectiveness, appropriateness and economic and socioeconomic benefit of such policy. Therefore, political priority for solutions to these challenges is needed to increase acceptability and enhance successful policy adoption and implementation of an SSBs tax.

### Strengths and limitations

Our findings should be interpreted in light of the strengths and limitations. The main strength of this review is that it is, to our knowledge, the first study that provides a systematic overview of the existing literature on political and public acceptability of an SSBs tax. The MMAT revealed all qualitative studies to have high levels of methodological quality (i.e. thirteen studies with an overall quality rating of four out of four criteria met, and four studies with an overall quality rating of three out of four criteria met), which increases confidence in our qualitative findings. The use of a mixed-method design further strengthens the findings of our review. Mixing methods “combines the power of stories and the power of numbers”, and is described to be useful in understanding a phenomenon (qualitative methods) as well as measure its magnitude (quantitative methods) [23].

The present review also has several limitations. Firstly, the search yielded few qualitative studies and no quantitative studies that investigated political acceptability of an SSBs tax, which may have been caused by the focus on scientific literature and not including policy documents. This could have resulted in an incomplete view of political acceptability. Notwithstanding, the qualitative synthesis provides important insights into political acceptability of the tax. Secondly, our findings may not be representative of all countries worldwide due to overrepresentation of studies conducted in the US, Australia and the UK, which indicates a need for studies in a wider range of countries. However, it is important to note that the qualitative synthesis of this review intended to generate an in-depth understanding of political and public acceptability of an SSBs tax worldwide and to explore beliefs that have implications for this acceptability rather than produce generalizable findings. Insufficient data were available to support the qualitative synthesis with quantitative estimates at national level (i.e. subgroup meta-analysis by countries). Generalization of our findings should therefore take into account national perceptions and circumstances. Moreover, insufficient data were available to estimate pooled proportions for the majority of subthemes that emerged from the qualitative synthesis. These subthemes should be measured in future research. Thirdly, although previous research indicates that acceptability of an SSBs tax varies among sociodemographic factors (e.g. age and educational level), subgroup meta-analysis was not performed because insufficient data were available. This may have contributed to the significant heterogeneity that was found across studies. Fourthly, only five studies investigated both political and public acceptability of an SSBs tax. Although we have judged qualitative studies on whether an appropriate consideration was given how their findings relate to researchers’ influence (e.g. through their interactions with participants) using the MMAT, we cannot exclude that observed differences between the findings of studies that only investigated political or public acceptability may have been caused by differences in researchers’ influence. Fifthly, to generate understanding of political and public acceptability of an SSBs tax data included in this review were cross-sectional. Future studies should explain what determined the beliefs identified in this review and explore potential solutions which might change those beliefs. In addition, future studies should investigate acceptability of an SSBs tax pre- and post-implementation to generate insights into the potential changes in beliefs related to the policy adoption and implementation of the tax. Sixthly, the MMAT revealed several included quantitative studies to have low levels of methodological quality (i.e. four studies with an overall quality rating of one out of four criteria met), which may have affected our estimates. For example, five quantitative studies were judged to have unrepresentative samples, which may have influenced our estimates. Future quantitative studies on political and public acceptability of an SSBs tax should use random sampling strategies, strive for representative samples and high response rates, as well as transparently report on those aspects. Finally, although political and public acceptability are important dimensions, they are only a part of several factors that could influence adoption and implementation of an SSBs tax. Therefore, we recommend further research to understand acceptability of other stakeholders (e.g. the industry, public health community and media), and other factors (e.g. feasibility) that could influence successful policy adoption and implementation.

## Conclusions

In conclusion, beliefs about effectiveness and cost-effectiveness, about appropriateness, about economic and socioeconomic benefit, about policy adoption, and public mistrust of the industry, government and public health experts have important implications for political and public acceptability of an SSBs tax. Our review provides several recommendations to consider to increase acceptability and enhance successful adoption and implementation of an SSBs tax: (i) address inconsistencies between beliefs about an SSBs tax and scientific literature, (ii) use raised revenue for health initiatives rather than for the general budget, (iii) communicate transparently about the true purpose of the tax, and (iv) generate political priority for solutions to the challenges to policy adoption and implementation.

## Additional file

Additional file 1: **Table S1a.** PubMed search (November 14th 2018). **Table S1b.** Embase search (November 14th 2018). **Table S1c.** Scopus (November 14th 2018). **Table S1d.** Web of Science (November 14th 2018). **Table S2a.** Methodological quality criteria from the Mixed Methods Appraisal Tool (MMAT). **Table S2b.** Quality appraisal of the studies included in the qualitative synthesis. **Table S2c.** Quality appraisal of the studies included in the quantitative synthesis. **Table S3.** Criteria for the extraction of proportions. (DOCX 23 kb)

## Data Availability

Not applicable.

## Abbreviations

MMAT: Mixed Methods Appraisal Tool
RCTs: Randomized controlled trials
SSBs: Sugar-sweetened beverages
WHO: World Health Organization
