# Supplementary material for: Political and public acceptability of a sugar-sweetened beverages tax: a mixed-method systematic review and meta-analysis
Source: Int J Behav Nutr Phys Act. 2019 Sep 4;16:78. doi: 10.1186/s12966-019-0843-0 (PMC6727579; doi:10.1186/s12966-019-0843-0)
Supplement: Supplementary file 1 — Table S1a. PubMed search (November 14th 2018). Table S1b. Embase search (November 14th 2018). Table S1c. Scopus (November 14th 2018). Table S1d. Web of Science (November 14th 2018). Table S2a. Methodological quality criteria from the Mixed Methods Appraisal Tool (MMAT). Table S2b. Quality appraisal of the studies included in the qualitative synthesis. Table S2c. Quality appraisal of the studies included in the quantitative synthesis. Table S3. Criteria for the extraction of proportions. (DOCX 23 kb) [file 12966_2019_843_MOESM1_ESM.docx]

SUPPLEMENTARY MATERIAL

**Table S1a.** PubMed search (November 14^th^ 2018).

| Search | Terms | Results |
| --- | --- | --- |
| 1 | "Carbonated Beverages"[Mesh] OR “Energy Drinks”[Mesh] OR soda[tiab] OR sodas[tiab] OR sugar*[tiab] OR ((drink[tiab] OR drinks[tiab] OR beverage*[tiab]) NOT alcohol[tiab]) OR ((drink[tiab] OR drinks[tiab] OR beverage*[tiab]) AND ("Sweetening Agents"[Mesh] OR "Nutritive Sweeteners"[Mesh] OR sugar*[tiab] OR sweeten*[tiab] OR carbonated[tiab] OR caloric[tiab] OR energy[tiab] OR soft[tiab])) | 146574 |
| 2 | “Taxes”[Mesh] OR “Fiscal policy”[Mesh] OR tax[tiab] OR taxes[tiab] OR taxing[tiab] OR taxation[tiab] OR price*[tiab] OR pricing[tiab] OR fiscal[tiab] OR financial[tiab] OR penalty[tiab] OR penalties[tiab] OR levy[tiab] OR levies[tiab] | 140318 |
| 3 | “Public Policy"[Mesh:NoExp] OR "Health Policy"[Mesh] OR "Nutrition Policy"[Mesh] OR "Decision Making"[Mesh] OR “Government”[Mesh] OR political[tiab] OR policy[tiab] OR policies[tiab] OR decisionmak*[tiab] OR decision mak*[tiab] OR government*[tiab] OR “Public Opinion”[Mesh] OR “Attitude”[Mesh] OR “Behavior”[Mesh] OR “Knowledge”[Mesh] OR “Perception”[Mesh] OR opinion*[tiab] OR attitude*[tiab] OR behavior*[tiab] OR behaviour*[tiab] OR knowledge[tiab] OR perception*[tiab] OR response*[tiab] OR view*[tiab] OR idea*[tiab] OR support[tiab] OR opposition[tiab] OR barrier*[tiab] OR accept*[tiab] OR argument*[tiab] OR implement*[tiab] OR feasibil*[tiab] OR adoption[tiab] OR motive*[tiab] OR judgment*[tiab] OR judgement*[tiab] OR perspective*[tiab] OR prefer*[tiab] OR pro-choice[tiab] | 7842882 |
|  | #1 AND #2 AND #3 | 1008 |

**Table S1b.** Embase search (November 14^th^ 2018).

| Search | Terms | Results |
| --- | --- | --- |
| 1 | 'carbonated beverage'/exp OR 'sweetened beverage'/exp OR 'energy drink'/exp OR 'soft drink'/exp OR soda:ab,ti,kw OR sodas:ab,ti,kw OR sugar*:ab,ti,kw OR ((drink:ab,ti,kw OR drinks:ab,ti,kw OR beverage*:ab,ti,kw) NOT alcohol:ab,ti,kw) OR ((drink:ab,ti,kw OR drinks:ab,ti,kw OR beverage*:ab,ti,kw) AND ('sweetening agent'/exp OR sugar*:ab,ti,kw OR sweeten*:ab,ti,kw OR carbonated:ab,ti,kw OR calosric:ab,ti,kw OR energy:ab,ti,kw OR soft:ab,ti,kw)) | 176556 |
| 2 | 'tax'/exp OR 'fiscal policy'/exp OR tax:ab,ti,kw OR taxes:ab,ti,kw OR taxing:ab,ti,kw OR taxation:ab,ti,kw OR price*:ab,ti,kw OR pricing:ab,ti,kw OR fiscal:ab,ti,kw OR financial:ab,ti,kw OR penalty:ab,ti,kw OR penalties:ab,ti,kw OR levy:ab,ti,kw OR levies:ab,ti,kw | 184741 |
| 3 | 'public policy'/de OR 'health care policy'/exp OR 'decision making'/exp OR 'government'/exp OR political:ab,ti,kw OR policy:ab,ti,kw OR policies:ab,ti,kw OR decisionmak*:ab,ti,kw OR ‘decision mak*’:ab,ti,kw OR government*:ab,ti,kw OR 'public opinion'/exp OR 'attitude'/exp OR 'behavior'/exp OR 'knowledge'/exp OR 'perception'/exp OR opinion*:ab,ti,kw OR attitude*:ab,ti,kw OR behavior*:ab,ti,kw OR behaviour*:ab,ti,kw OR knowledge:ab,ti,kw OR perception*:ab,ti,kw OR response*:ab,ti,kw OR view*:ab,ti,kw OR idea*:ab,ti,kw OR support:ab,ti,kw OR opposition:ab,ti,kw OR barrier*:ab,ti,kw OR accept*:ab,ti,kw OR argument*:ab,ti,kw OR implement*:ab,ti,kw OR feasibil*:ab,ti,kw OR adoption:ab,ti,kw OR motive*:ab,ti,kw OR judgment*:ab,ti,kw OR judgement*:ab,ti,kw OR perspective*:ab,ti,kw OR prefer*:ab,ti,kw OR ‘pro-choice’:ab,ti,kw | 10391021 |
|  | #1 AND #2 AND #3 | 1364 |

**Table S1c.** Scopus (November 14^th^ 2018).

| Search | Terms | Results |
| --- | --- | --- |
| 1 | TITLE-ABS-KEY(soda OR sodas OR sugar* OR ((drink OR drinks OR beverage*) AND NOT alcohol) OR ((drink OR drinks OR beverage*) AND (sugar* OR sweeten* OR carbonated OR caloric OR energy OR soft))) | 378041 |
| 2 | TITLE-ABS-KEY(tax OR taxes OR taxing OR taxation OR price* OR pricing OR fiscal OR financial OR penalty OR penalties OR levy OR levies) | 1045238 |
| 3 | TITLE-ABS-KEY(political OR policy OR policies OR decisionmak* OR “decision mak*” OR government* OR opinion* OR attitude* OR behavior* OR behaviour* OR knowledge OR perception* OR response* OR view* OR idea* OR support OR opposition OR barrier* OR accept* OR argument* OR implement* OR feasibil* OR adoption OR motive* OR judgment* OR judgement* OR perspective* OR prefer* OR “pro-choice”) | 21968180 |
|  | #1 AND #2 AND #3 | 3745 |

**Table S1d.** Web of Science (November 14^th^ 2018).

| Search | Terms | Results |
| --- | --- | --- |
| 1 | TS= (“soda” OR “sodas” OR sugar* OR ((“drink” OR “drinks” OR beverage*) NOT “alcohol”) OR ((“drink” OR “drinks” OR beverage*) AND (sugar* OR sweeten* OR “carbonated” OR “caloric” OR “energy” OR “soft”))) | 268519 |
| 2 | TS=(“tax” OR “taxes” OR “taxing” OR “taxation” OR price* OR “pricing” OR “fiscal” OR “financial” OR “penalty” OR “penalties” OR “levy” OR “levies”) | 536806 |
| 3 | TS=(“political” OR “policy” OR “policies” OR decisionmak* OR “decision mak*” OR government* OR opinion* OR attitude* OR behavior* OR behaviour* OR “knowledge” OR perception* OR response* OR view* OR idea* OR “support” OR “opposition” OR barrier* OR accept* OR argument* OR implement* OR feasibil* OR “adoption” OR motive* OR judgment* OR judgement* OR perspective* OR prefer* OR “pro-choice”) | 12399149 |
|  | #1 AND #2 AND #3 | 2205 |

**Table S2a**. Methodological quality criteria from the Mixed Methods Appraisal Tool (MMAT)

| Study design | Criteria* |
| --- | --- |
| All designs (screening questions) | - Are there clear qualitative and quantitative research questions (or objectives), or a clear mixed methods question (or objective)? - Do the collected data allow address the research question (objective)? |
| Qualitative study or qualitative component of a mixed-method study | 1. Are the sources of qualitative data (archives, documents, informants, observations) relevant to address the research question (objective)? 2. Is the process for analyzing qualitative data relevant to address the research question (objective)? 3. Is appropriate consideration given to how findings relate to the context, e.g., the setting, in which the data were collected? 4. Is appropriate consideration given to how findings relate to researchers’ influence, e.g., through their interactions with participants? |
| Quantitative descriptive study or quantitative component of a mixed-method study | 1. Is the sampling strategy relevant to address the quantitative research question (quantitative aspect of the mixed methods question)? 2. Is the sample representative of the population understudy? 3. Are measurements appropriate (clear origin, or validity known, or standard instrument)? 4. Is there an acceptable response rate (60% or above)? |

*For each methodological quality criterium answer Yes, No, or Can’t tell. A tutorial with definitions and examples for each criterium is available in the MMAT 2011: http://mixedmethodsappraisaltoolpublic.pbworks.com

**Table S2b**. Quality appraisal of the studies included in the qualitative synthesis.

| Author, year | Overall quality | 1 | 2 | 3 | 4 |
| --- | --- | --- | --- | --- | --- |
| Chan, 2009 | **** | Yes | Yes | Yes | Yes |
| Francis, 2017 | **** | Yes | Yes | Yes | Yes |
| Giabbanelli, 2016 | **** | Yes | Yes | Yes | Yes |
| Isett, 2015 | **** | Yes | Yes | Yes | Yes |
| Krukowski, 2016 | **** | Yes | Yes | Yes | Yes |
| Lloyd-Williams, 2014 | **** | Yes | Yes | Yes | Yes |
| Moise, 2011 | **** | Yes | Yes | Yes | Yes |
| Moretto, 2014 | **** | Yes | Yes | Yes | Yes |
| Nixon, 2015 | *** | Yes | Yes | Yes | No |
| Ortega-Avila, 2018 | **** | Yes | Yes | Yes | Yes |
| Purtle, 2018 | *** | Yes | Yes | Yes | No |
| Signal, 2018 | **** | Yes | Yes | Yes | Yes |
| Swift, 2018 | **** | Yes | Yes | Yes | Yes |
| Tamir, 2018 | *** | Yes | Yes | Yes | No |
| Thomas-Meyer, 2017 | **** | Yes | Yes | Yes | Yes |
| Thow, 2011 | *** | Yes | Yes | Yes | No |
| Visram, 2017 | **** | Yes | Yes | Yes | Yes |

**Table S2c**. Quality appraisal of the studies included in the quantitative synthesis.

| Author, year (reference) | Overall quality | 1 | 2 | 3 | 4 |
| --- | --- | --- | --- | --- | --- |
| Álvarez-Sánchez, 2018 | **** | Yes | Yes | Yes | Yes |
| Barry, 2013 | *** | Yes | Yes | Yes | No |
| Brock, 2017 | *** | Yes | Yes | Yes | No |
| Comans, 2017 | * | Can’t tell | Can’t tell | Yes | No |
| Curry, 2018 | *** | Yes | Yes | Yes | No |
| Donaldson, 2015 | *** | Yes | Yes | Yes | Can’t tell |
| Farrell, 2018 | *** | Yes | Yes | Yes | No |
| Gollust, 2014 | ** | Yes | Can’t tell | Yes | No |
| Gollust, 2017 | ** | Yes | No | Yes | Can’t tell |
| Julia, 2015 | *** | Yes | Yes | Yes | No |
| Moretto, 2014 | * | No | No | Yes | No |
| Morley, 2012 | *** | Yes | Yes | Yes | No |
| Niederdeppe, 2014 | *** | Yes | Yes | Yes | No |
| Petrescu, UK, 2016 | ** | Can’t tell | Yes | Yes | Can’t tell |
| Petrescu, US, 2016 | ** | Can’t tell | Yes | Yes | Can’t tell |
| Rivard, 2012 | *** | Yes | Yes | Yes | No |
| Roh, Study 2, 2016 | * | No | No | Yes | Can’t tell |
| Roh, Study 3, 2016 | *** | Yes | Yes | Yes | No |
| Sainsbury, 2018 | *** | Yes | Yes | Yes | Can’t tell |
| Scully, 2017 | * | No | No | Yes | No |
| Simon, 2014 | **** | Yes | Yes | Yes | Yes |
| Swift, 2018 | *** | Yes | Yes | Yes | Can’t tell |
| Tabak, 2013 | ** | Yes | No | Yes | No |
| Wolfson, Policy Support Survey, 2015 | *** | Yes | Yes | Yes | No |

**Table S3**. Criteria for the extraction of proportions.

| Response option | Method |
| --- | --- |
| Dichotomous (yes/no) | The proportion of participants choosing either ‘yes’ or ‘no’, depending on which indicated support |
| Yes/unsure/no | The proportion of participants choosing either ‘yes’ or ‘no’, depending on which indicated support |
| 4-point scale | The proportion of participants choosing 3 or 4 OR the proportion of participants choosing 1 or 2, depending on which indicated support |
| 5-point scale | The proportion of participants choosing 4 or 5 OR the proportion of participants choosing 1 or 2, depending on which indicated support |
| 7-point scale | The proportion of participants choosing within the range of 5-7 OR the proportion of participants choosing 1-3, depending on which indicated support |
| 10-point scale | The proportion of participants responding within the range of 1-4 OR the proportion of participants responding within the range of 7-10, depending on which indicated support |
| 100-point scale | The proportion of participants responding within the range of 1-40 OR the proportion of participants responding within the range of 70-100, depending on which indicated support |

Adapted from Sheals *et al*.: https://www.ncbi.nlm.nih.gov/pmc/articles/PMC5025720/
